# Connecting gut microbiomes and short chain fatty acids with the serotonergic system and behavior in *Gallus gallus* and other avian species

**DOI:** 10.3389/fphys.2022.1035538

**Published:** 2022-11-02

**Authors:** Vidya V. Jadhav, Jian Han, Yewande Fasina, Scott H. Harrison

**Affiliations:** ^1^ Department of Biology, North Carolina Agricultural and Technical State University, Greensboro, NC, United States; ^2^ Department of Animal Sciences, North Carolina Agricultural and Technical State University, Greensboro, NC, United States

**Keywords:** gut microbiome, gut‐brain axis, short chain fatty acids, serotonin, chicken, avians, behavior, translational science

## Abstract

The chicken gastrointestinal tract has a diverse microbial community. There is increasing evidence for how this gut microbiome affects specific molecular pathways and the overall physiology, nervous system and behavior of the chicken host organism due to a growing number of studies investigating conditions such as host diet, antibiotics, probiotics, and germ-free and germ-reduced models. Systems-level investigations have revealed a network of microbiome-related interactions between the gut and state of health and behavior in chickens and other animals. While some microbial symbionts are crucial for maintaining stability and normal host physiology, there can also be dysbiosis, disruptions to nutrient flow, and other outcomes of dysregulation and disease. Likewise, alteration of the gut microbiome is found for chickens exhibiting differences in feather pecking (FP) behavior and this alteration is suspected to be responsible for behavioral change. In chickens and other organisms, serotonin is a chief neuromodulator that links gut microbes to the host brain as microbes modulate the serotonin secreted by the host’s own intestinal enterochromaffin cells which can stimulate the central nervous system via the vagus nerve. A substantial part of the serotonergic network is conserved across birds and mammals. Broader investigations of multiple species and subsequent cross-comparisons may help to explore general functionality of this ancient system and its increasingly apparent central role in the gut-brain axis of vertebrates. Dysfunctional behavioral phenotypes from the serotonergic system moreover occur in both birds and mammals with, for example, FP in chickens and depression in humans. Recent studies of the intestine as a major site of serotonin synthesis have been identifying routes by which gut microbial metabolites regulate the chicken serotonergic system. This review in particular highlights the influence of gut microbial metabolite short chain fatty acids (SCFAs) on the serotonergic system. The role of SCFAs in physiological and brain disorders may be considerable because of their ability to cross intestinal as well as the blood-brain barriers, leading to influences on the serotonergic system via binding to receptors and epigenetic modulations. Examinations of these mechanisms may translate into a more general understanding of serotonergic system development within chickens and other avians.

## Introduction to the chicken gut-microbiome-brain axis

Chickens are an important source of food in the human diet worldwide, and the poultry industry is one of the fastest-growing fields in agriculture (Nkukwana, 2019). Ongoing studies surrounding chicken husbandry and physiology have generated substantial amounts of knowledge regarding the chicken gut-microbiome-brain axis. The diverse chicken gut microbiome, for instance, is now known to have strong effects on the feed conversion ratio impacting growth and health (Stanley et al., 2012), early stages of immune system development (Schokker et al., 2017), resistance to enteric pathogens (Feng et al., 2010), and behavior (Kraimi et al., 2019a).

There are multiple routes to how host physiology and molecular processes interact with different gut microbial varieties and associated microbial metabolites. Management practices like overcrowding in cages, high temperature, and rough transportation all of which exert stress on chickens (Virden and Kidd, 2009; Sanchez-Casanova et al., 2019). These stressors in chicken affect gut microbial community composition. This is evident by studies involving external environmental stressors and studies administering corticosterone (Calefi et al., 2016; Noguera et al., 2018; Zaytsoff et al., 2020). The changes in the gut microbiome composition may be induced by the CNS via the sympathoadrenal system and the hypothalamic-pituitary-adrenal (HPA) axis (Villageliũ and Lyte, 2017). Decades of research have shown the effect of these stressors on host serotonin synthesis (Chaouloff et al., 1999). Other neurochemicals along with serotonin have been documented in the broiler chicken intestinal track with their levels being altered during a stressed condition (Dennis, 2009; Lyte et al., 2022). The systemic circulation of neurochemicals in chickens has been found to affect general physiology (Denbow et al., 1983; Chapman et al., 2008) and the immune system (Borsoi et al., 2015), as well as the gut and growth of different bacterial species including pathogens (Lyte and Ernst, 1992; Bailey et al., 1999; Freestone et al., 2008; Truccollo et al., 2020; Lyte et al., 2021a). Gut microbiota are furthermore known to produce and stimulate host neurotransmitter synthesis, with these effects found to ultimately influence host physiology and behavior (Beaver and Wostmann, 1962; Reigstad et al., 2015; Van Staaveren et al., 2021). Such a bidirectional relationship between microbiomes and neurochemistry was recently demonstrated in a Japanese quail model where management stress response led to changes in microbial composition, with the effect of gut microbes on tissue serotonin concentration outside the gut being also observed (Lyte et al., 2021b).

Serotonin levels in the gut are influenced by gut microbes as has been demonstrated by pioneering studies comparing conventional and germ-free chick models (Phillips et al., 1961; Beaver and Wostmann, 1962). Serotonin is a major neurotransmitter regulating aggression in chicken (Dennis, 2009). Chickens may cope with stress by exhibiting aggressive behavior such as, for example, aggressive FP (Cheng and Muir, 2007; Van Staaveren and Harlander, 2020). FP birds harm not only themselves but also other birds by pecking and pulling their feathers leads to decreased performance of birds and loss to the poultry industry (Jensen et al., 2005). Several studies also indicate a regulatory role of gut microbes in the gut-brain axis that includes probiotic modulations that mitigate aggressive behavior in birds (Abdel-Azeem, 2013; Cheng et al., 2019; Mindus et al., 2021). Dietary modulations of gut microbiota have been found to overall improve chicken behavior and overall health (Dixon and Nicol, 2008; Pan and Yu, 2014). The gut microbial modulation could therefore have considerable value with respect to common challenges with chicken health and husbandry. Beyond discovering effects of microbiomes on chickens, a translational objective is to evaluate whether advantages of chickens as a model organism and underlying mechanisms of the chicken gut-microbiome-brain axis would help to inform understanding and investigation of the gut-microbiome-brain axis in humans. For instance, with humans, stress and diet substantially alter gut microbial ecosystems with varying impacts on human health (Singh et al., 2017; Gubert et al., 2020). Common mechanisms surrounding the gut-brain axis in humans and chickens involve the serotonergic system being modulated by conditions of stress (Leonard, 2005). The impact of gut microbes on the serotonergic system and behavior have been closely linked with phenotypes of FP in chicken and depression in humans (Cheng et al., 2019; Huang and Wu, 2021).

For various animals, including chickens and humans, serotonin is mainly synthesized by serotonergic neurons in brain and intestinal enterochromaffin cells (Parent, 1981). The large portion of serotonin in the body is produced by intestinal enterochromaffin cells and production is stimulated by gut microbial metabolites like SCFAs (Gershon, 2013; Reigstad et al., 2015). Some of the more abundant microbial SCFAs, butyrate and acetate, induce dramatic shifts of expression for the rate-limiting enzyme, Tryptophan hydroxylase 1 (Tph1), which is associated with mucosal serotonin synthesis by intestinal enterochromaffin cells (Côté et al., 2003; Reigstad et al., 2015). This review provides a report and synthesis of current molecular and physiological findings surrounding how the serotonergic system and behavior relate to gut microbiota and SCFAs in chickens. This review, in addition, critically evaluates the use of chicken as an animal model that may help influence and guide the study of the gut-microbiome-brain axis in humans as would relate to SCFAs and the serotonergic system.

## Chicken gut microbiota and potential function

The gastrointestinal (GI) tract of chicken is inhabited by a complex and dynamic microbial community that is established during hatching and initial period of exposure to the environment, stabilizing later in life. Chickens hatched within hatcheries receive microbes from environmental flora (Stanley et al., 2013; Volf et al., 2021). This microbiome undergoes dramatic changes, overall expanding throughout the life of a chicken, leading to an adult chicken GI tract having trillions of bacteria, representing more than 600 bacterial species (Apajalahti et al., 2004; Apajalahti & Kettunen, 2006; Borda-Molina et al., 2018). Similar to what has been found for human gut microbiota, analyses of broiler and layer chicken gut microbiota have identified *Proteobacteria*, *Bacteroidetes*, and *Firmicutes* as the more abundant phyla. Other phyla, such as *Actinobacteria*, while less abundant, are consistently found as well (Qin et al., 2010; Li et al., 2014; Tong et al., 2017; Mandal et al., 2020).

In the chicken GI tract, the cecum is a major anatomical location with higher microbial diversity and metabolism (Sergeant et al., 2014; Polansky et al., 2016). Recent metagenomic analysis of the chicken cecum has identified 42 novel genera, 40 of which are of the taxonomic class *Clostridia* which is observed in high abundance in the ceca. More prevalent taxonomic orders within the *Clostridia* class are *Oscillospirales* and *Lachnospirales* (Glendinning et al., 2020). At the family level, the cecum encompasses *Clostridiaceae*, *Bacteroidaceae*, *Lactobacillaceae*, and SCFA butyrate producing *Lachnospiraceae* families (Witzig et al., 2015). Analyses of the chicken microbiome found in the cecum have helped to identify new gut microbes and unravel their functionality. For instance, it has revealed those microbes having genetic material that encodes polysaccharide and numerous oligosaccharide-degrading enzymes. The degrading of polysaccharides occurs in large part due to lineages belonging to the taxonomic classes *Actinobacteria*, *Clostridia,* and *Bacteroidia*. Genes involved in SCFA (acetate and butyrate) production have furthermore been identified, with most of these genes and their associated functions occurring for lineages that belong to the *Firmicutes* and *Bacteroidetes* phyla (Sergeant et al., 2014).

The metabolic capacity and overall colonization pattern of gut microbes lead to various health benefits and behavioral outcomes for chicken and other avian species. The main source of carbon and energy for the microbes in the lower intestine comes from undigested complex dietary carbohydrates and starch (Cummings and Macfarlane, 1991). Moreover, plant-based poultry diets have a large amount of non-starch polysaccharides (NSP) (Józefiak et al., 2004; Raza et al., 2019). Fermentation of undigested food by gut microbes in the cecum and colon produces SCFAs (van der Wielen et al., 2000), which benefit the host by providing a source of energy, stimulating gut epithelial cell proliferation, and by lowering the colon pH to help prevent secondary bile production (Sakata, 1997). Some beneficial gut microbes are known to protect the intestine against colonization by pathogenic bacteria such as *Salmonella* spp. (Nurmi and Rantala, 1973). In addition, gut bacteria produce and sometimes metabolize various neurochemicals like serotonin, essential amino acids like tryptophan, vitamins, and antimicrobial compounds (Jeurissen et al., 2002; Yanofsky, 2007; Lyte, 2011; Kogut, 2019). Much of the same has been generally found for humans (Rowland et al., 2018). Gut bacteria have an overall regulatory impact on the gut-brain axis leading to behavioral changes as well (Cryan and Dinan, 2012; Arneth, 2018). A dietary study of great tits, being provided an insect diet versus a seed diet, showed compositional change in the gut microbiome occurring in parallel to reduced problem-solving skills for birds fed the insect diet (Davidson et al., 2020).

## Effect of gut microbiota on cognition and behavior

Domestic chickens are the most common and widely used species of poultry in agriculture and are a domesticated breed of red junglefowl (*G. gallus*) (Siegel et al., 1992; Yamashita et al., 1994). Despite many effects of selective breeding, domestic chickens retain cognitive and behavioral similarity to their ancestors. Both wild and domestic chickens follow a similar social structure and behavior of interaction within their populations and have complex cognitive ability, along with emotional and communicative behavior (Appleby et al., 2004). Hens and chicks are in the center of a domestic chicken community whereas roosters live independently and protect hen and chicks in the group. Chickens communicate information regarding territory, mating, nesting, distress, danger or fear, contentment, and food discovery with the help of 30 distinct vocalizations (Appleby et al., 2004). Findings regarding fear response show complex emotional behavior which is accompanied by physiological reactions like fever that can also be found with humans (Cabanac and Aizawa, 2000).

For how gut microbiomes and their metabolic products connect dynamics within the gut to the brain, resulting in effects on behavior, this is being studied as an applied area of research that may considerably improve our understanding of human health and animal behavior and wellness. It has indeed been possible to adjust the microbiome toward positive behavioral outcomes with, for example, supplementation with *Lactobacillus rhamnosus* between 19 and 26 weeks of age being found to reduce FP in chickens (Mindus et al., 2021) (Table 1). Gut microbial composition changes have shown the potential to aid mammals in their adaptation to stress as well (Boonstra, 2005). Biomedical findings arising mainly from studies on humans and mice have found gut microbial-derived products like neurotransmitters, SCFAs, indoles, bile acids, choline metabolites, lactate, and vitamins to have general effects across animal host physiology (Krautkramer et al., 2021). Broad-ranging impacts between microbiomes and behavior have been found in chickens, quail, and turkey (Table 1). A recent study of Japanese quail has demonstrated how emotional reactivity can be influenced by gut microbiota transfers that alter taxa of the *Firmicutes* phylum (Kraimi et al., 2018; Kraimi et al., 2019b). Changes in abundance for the *Firmicutes* phylum have also been associated with stress, anxiety, or depression (Bailey et al., 2011; Jiang et al., 2015). In a similar study in turkey, probiotic administration has been found to reduce distress calls and agonistic behavior in birds (Abdel-Azeem, 2013). On the contrary, the prolonged deprivation of natural bird behaviors like foraging, nesting, perching, and dust-bathing is believed to affect brain function and lower gut microbial diversity (Chen et al., 2019).

**TABLE 1:** Studies investigating effects of gut microbiota interventions on bird behavior.

Study details	Bird species	Behavioral outcome	Findings	References
Ingesion of *L. rhamnosus*	White Leghorn, laying hens; Selected HFP, LFP lines	Reduced stress induced FP	Increased T cell population of spleen and the cecal tonsils Limited cecal microbial dysbiosis	Mindus et al. (2021)
FMT during early life from aged donor	Healthy commercial broilers	FM from adult chickens improves fearfulness in chicks	FMT administration might improve the physiology and behavior of chickens	Yan et al. (2021)
Early life FMT from HFP or LFP adults	White Leghorn birds Selected HFP, LFP lines	FMT influenced FP behavior; Homologous FMT resulted in reduced fearfulness	FMT had immediate and long-term effects on behavior and immune characteristics and peripheral serotonin	van der Eijk et al. (2020)
FM transfer from 13 weeks old adult female quails in GF Chicks	Japanese quails from quail line selected for high (E+) and low (E−) emotional reactivity	GM from (E−) quails in GF chicks reduced emotional reactivity in early life	Change in the GM composition in treatment groups associated with behavioral modification	Kraimi et al. (2019)
GF quails compared to quails with FM from adult female quails	Japanese quails	GF quails showed reduced emotional reactivity compared to quails with gut microbiota	Absence of gut microbiota reduces emotional reactivity in Japanese quails with no effect on growth	Kraimi et al. (2018)
Effect of heat stress and *or Clostridium perfringens* infection	Broiler chickens	*C. perfringens* infection decreased the frequency of feeding, walking, FP and standing; Increased the frequency of SB behavior	Showed links among degree of intestinal lesions, behavioral outcomes, brain activity, and serum levels of corticosterone	Calefi et al. (2016)
Administration of probiotic spores of *Bacillus amyloliquefaciens*	Turkey poults	Probiotics administration increased the feeding frequency and decreased distress call and aggressive behaviors		Abdel-Azeem (2013)

CR, cage rearing; FR, free-range; FMT, fecal microbiota transplantation; FM, fecal microbiota; FP, feather pecking; SB, sickness behavior; GF, germ free; GM, gut microbiota; HFP, high feather pecking; LFP, low feather pecking; SCFAs, short chain fatty acids.

There have been some initial studies on the association of gut microbial metabolites with chicken behavior. A study conducted by Meyer et al. (2013) investigated differences in gut microbial metabolites in high and low FP chickens. The study analyzed gut metabolites like biogenic amines, SCFAs, ammonia, and lactate. Total SCFAs were elevated in high FP birds due to the utilization of ingested feathers by cecal microbes (Meyer et al., 2013). While chicken gut microbial composition is increasingly studied for microbial diversity and microbial modulations that influence poultry production (Grond et al., 2018), there remains a dearth of metabolomic and functional studies illustrating the effect of microbial metabolites on host physiology and behavior. As shown in Table 1, not all studies evaluate for behavioral outcomes along with both microbial and metabolite-related outcomes. These studies also varied in terms of ages studied, with some only lasting for a few weeks (Abdel-Azeem, 2013; Calefi et al., 2016; Kraimi et al., 2018) and others continuing for two or more months (van der Eijk, et al., 2020; Mindus et al., 2021; Yan et al., 2021). Future studies are needed to evaluate dynamics across potentially interconnected microbial and metabolite-related outcomes.

## The serotonergic system

Serotonin is an important neurotransmitter that connects the gut-brain axis and exists ubiquitously across diverse biological systems, including for vertebrates, invertebrates, and some plants (phytoserotonin) (Smith, 1971). Central serotonin has been found to regulate temperature (Freeman, 1979), appetite, sleep, and energy metabolism (Lv and Liu, 2017; Hillman et al., 1980). Serotonin is also associated with cognition and behavior across the animal kingdom (Bacqué -Cazenave et al., 2020), which makes the serotonin system a potential target for treating behavioral problems (Nishizawa et al., 1997).

Peripheral serotonin acts as hormone and improves nutrient absorption, and regulates GI motility, pancreatic secretion and peristaltic reflex (Martin et al., 1993; Li et al., 2001). It participates in multiple physiological functions through the diverse receptors it binds to, including vasoconstriction and dilation (Rapport et al., 1949), adipogenesis in white adipose tissue (WAT), muscle, and liver glucose uptake (Namkung et al., 2015). Serotonin modulates insulin secretion and the immune system (Cataldo Bascunan et al., 2019). Within the intestine, serotonin acts as a pro-inflammatory as well as anti-inflammatory signaling molecule (Bischoff et al., 2009). Pro-inflammatory signaling is studied in serotonin transporter-knockout mice which exacerbates experimental GI inflammatory disease through activating 5-HT7 receptors expressed by dendritic cells (Bischoff et al., 2009; Kim et al., 2013). However, serotonin is also involved in anti-inflammatory signaling via epithelial 5-HT4 receptor activation, reducing colon inflammation in mice (Spohn et al., 2016).

### Central and peripheral serotonin system

Central serotonergic neurons are located in dorsal raphe and median raphe nuclei that are present in the midline of the brainstem (Puelles et al., 2018; Fujita et al., 2022). These neurons occupy most central nervous system regions with their projections (Reiner, 2001; Matragrano et al., 2012; García-González et al., 2017). As has been found in humans, chickens and other animals, serotonin is synthesized from its precursor tryptophan by the rate-limiting enzyme tryptophan hydroxylase 2 (Tph2) in the serotonergic neurons of the brain (Böhm et al., 1979; Fujita et al., 2022; Sako et al., 1986), while peripheral serotonin is synthesized by its isoform Tph1 (Walther et al., 2003). Cofactors (Fe^2+^), co-substrates (O_2_ and BH_4_) and stress hormones are also activators of Tph (i.e., Tph1 or Tph2). Sustained tryptophan hydroxylase activity influences the firing rate of serotonergic neurons (Maximino, 2012). Furthermore, tryptophan is an essential amino acid derived from the diet. Tph converts L-tryptophan into 5-hydroxytryptophan (5-HTP) which transforms into serotonin, 5-hydroxytryptamine (5-HT), by the action of aromatic L-amino acid decarboxylase (Leathwood, 1987). Serotonin has a very short half-life in the brain (Brodie and Reid, 1968). Active serotonin gets transported to the synaptic space while inactive serotonin is metabolized in and outside the cell. The enzyme monoamine oxidase A (MAO-A), located in the outer mitochondrial membrane of the neuron, deaminates or metabolizes 5-HT into 5-hydroxy-indol-acetaldehyde, which is then oxidized into urinary metabolite 5-hydroxy-indole-acetic acid (5-HIAA), a urinary marker of serotonin synthesis (Kuhn and Hasegawa, 2020). Disruptions to this 5-HT metabolism, mainly as regards 5-HIAA, is associated with aggressive behavior in mammals as well as birds (Coccaro et al., 2010; Kops et al., 2013). In the brain, high tryptophan levels increase the production of serotonin (Fernstrom and Wurtman, 1971). The brain receives peripheral tryptophan through active transportation across the blood-brain barrier, where tryptophan has to compete with tyrosine and other branched-chain amino acids for transport (Fernstrom and Fernstrom, 1995; Fernstrom and Fernstrom, 2007).

In the case of serotonergic transmission, synthesized neuronal serotonin is released from presynaptic neurons into the synaptic space through vesicle transport. Upon release, these molecules bind to serotonin receptors in the postsynaptic membrane and transmit signals to different brain projection areas (Millan et al., 2008). The excess serotonin in the synaptic space is bound to by the serotonin reuptake transport (SERT) membrane protein of presynaptic neurons (Krause et al., 2017). After reuptake in the raphe neuron, inactive serotonin is degraded by monoamine oxidase (MAO) (Borue et al., 2007). Binding of synaptic as well as peripheral serotonin to receptors modulate the central and peripheral function of serotonergic neurons and thus influence behavior. There are 14 serotonin receptor proteins identified in mammals and in poultry birds with varying distributions in the brain as well as peripheral regions (Banerjee et al., 2007; Stępińska et al., 2015).

Presence of serotonin in chicken GI track has been known for decades (Phillips et al., 1961), as has been known how enterochromaffin cells are distributed throughout the avian gut (Rawdon 1984). Apart from enterochromaffin cells, peripheral serotonin is synthesized by serotonergic neurons from the enteric nervous system (ENS) (Neuhuber and Worl, 2018). Out of these sites, enterochromaffin cells in the gut synthesize most of total body serotonin. A recent study providing concentration of neurotransmitters in the GI track of broiler chicken reported serotonin and 5-HIAA levels in tissue as well as luminal content at varying bird ages (Lyte et al., 2022). The tissue serotonin levels in jejunum, ileum, and cecum are higher than the luminal content levels at varying ages. Moreover, the luminal serotonin levels at jejunum, ileum, and cecum regions are not age dependent. This may indicate increased synthesis of serotonin in these regions.

Blood thrombocytes in birds store the serotonin produced (Maurer-Spurej, 2005), and the level of serotonin in the blood is strongly dependent upon its synthesis in the gut (Meyer et al., 1973). Upon release into the gut wall, serotonin acts as a luminal signal transducer to the central nervous system via intrinsic and extrinsic primary afferent neurons (vagal afferent neurons) of enteric nervous system (Li et al., 2000; Gershon and Tack, 2007). These afferent neurons receive and transmit physical as well as chemical stimuli to CNS initiated by enterochromaffin cells and immune cells. The enteric nervous system is an intrinsic system of the GI track. It is composed of neurons and glial cells that innervate the intestine and regulate GI motility, absorption, and fluid secretion (Doyle et al., 2004). Non-neuronal serotonin activates intrinsic primary afferent neurons of ENS through 5-HT1P receptor and mediates gut peristaltic and secretory reflexes, while the activation of the 5-HT3 receptor of extrinsic nerves communicates distress and other signals to the CNS (Gershon and Tack, 2007). Serotonin released outside the gut epithelium also activate the 5-HT4 receptor in the ENS and induce neuroprotective and neurogenerative effect (Liu et al., 2009). Serotonin produced by serotonergic neurons in the ENS influences gut motility and development of enteric neurons, and serotonin furthermore modulates the immune system (Neuhuber and Worl, 2018). However, there is less knowledge about functioning of these receptors in avian species (Stępińska et al., 2015).

### Similarity between the avian and mammalian serotonin system

Serotonin is an ancient and highly conserved biomolecule in the vertebrate species found to be localized in the raphe system and reticular nucleus (Challet et al., 1996; Hay-Schmidt, 2000). The serotonin system, including serotonin, 5-HT receptor structure and function, and serotonin transporter, is well-conserved across diverse vertebrates (Bubak et al., 2020). Distribution of serotonin in vertebrate brains has been studied decades ago and is found to coincide with expectations of phylogeny. A comparative study of serotonin and catecholamines distribution by Bogdanski et al. (1963) found occurrence of these amines in mammals and lower vertebrates, including fish and birds. In vertebrates, serotonin exhibits inhibitory action on aggressive behavior as has been observed across diverse animals. Autoradiography of neurotransmitter receptors in a brain basal ganglion in pigeon, rat and human brain have shown similarity in distribution. This includes the 5-HT1B receptor subtype in the globus pallidus (GP) region of basal ganglia which regulates the release of neurotransmitters including serotonin (Dietl and Palacios, 1988; Sari, 2004).

Anatomical structure of the serotonergic system is similar across different vertebrates, but levels of molecular expression and physiologic development do vary. A study reported the serotonin to catecholamine ratio to be 1.1:1 in rats while a 2:1 ratio has been reported in birds (Bogdanski et al., 1963). The anatomical distribution of monoamine-producing neurons in the avian brain has shown this cell population to occur in the hypothalamus (located below the thalamus) and lateral presence in tegmentum (the ventral part of the midbrain). Similar lateralization is also observed in mammals (Fuxe and Ljunggren, 1965; Dubé and Parent, 1981). Immunohistochemical and immunohistofluorescence techniques have been used to study distribution of serotonin fibers and terminals in pigeon brains and have found similarity in pattern as compared to mammals. Similar to the mammals in birds, serotonergic neurons in the midbrain tegmentum have shown descending projections towards the spinal cord whereas ascending projections towards prosencephalon (the future forebrain/cerebrum). The projection size is greater however in mammals than in birds (Challet et al., 1996).

### Gut microbes in serotonergic system development in avians and mammals

Diverse gut microbes acquired since birth influence neural pathways and CNS signaling, thus contributing to an organism’s systems-level development. This specific influence has been studied with various germ-free (GF) animal models (Smith, 2015). Developmental effect of gut microbiota on serotonergic system has been studied in a GF mouse model where chronic absence of microflora elevates striatal 5-HT turnover (Heijtz et al., 2011). Similar results have confirmed this in another study where, observed elevated hippocampal 5-HT and 5-HIAA levels did not change after restoring microbiota in later life. GF animals also exhibit abnormally reduced levels of anxiety which can be restored on GI microbiota transfer. This suggests a crucial role of intestinal microbes in influencing the central serotonin system (Clarke et al., 2013).

Gut microbiota are also known to play an important role in immune system and endocrine system development which are essential elements of CNS signaling. A recent GF study of mice has highlighted the impact of gut microbes on microglial cell maturation and activation where absence of microbes leads to microglial defects affecting innate immune response. This study found, in particular, microbial SCFAs to be a regulator of microglial homeostasis (Erny et al., 2015). Microglial cells have been recently studied as well for their interaction with serotonin and have had reported effects contributing to brain maturation (D’Andrea et al., 2020; Kolodziejczak et al., 2015). Another GF mouse study has shown gut microbes to influence adult ENS maturation through release of serotonin which further activates 5-HT4 receptor in ENS associated with adult neurogenesis and neuroprotection. The study demonstrated the difference in ENS anatomy in GF and with microbiota transfer models influencing intestinal function (De Vadder et al., 2018).

In the case of chickens, Beaver and Wostmann (1962) studied the influence of gut microbes on intestinal serotonin synthesis and observed reduced intestinal 5-HT levels in conventional chicken compared to germ free model. The influence of gut microbes on serotonin system development has been studied in the context of FP behavior whereas the serotonergic pathway is suspected to contribute to FP. The influence of gut microbiota on the serotonergic system and bird behavior has been studied by early life microbiota transplantation in hens selected for high and low FP. The investigation after 15 weeks of treatment observed variation in peripheral serotonin levels in low FP lines (Van der Eijk et al., 2020). There is another investigation on central serotonin turnover in 28 days-old chicks. Lower serotonin turnover was found for high FP chicks, but this study did not observe an influence of gut microbes (Van Hierden et al., 2002). The regulatory influence of gut microbes on peripheral serotonin system has been established in birds, mice, rats, and humans as well, including for instances specific to disease (Phillips et al., 1962; Böhm et al., 1979; Uribe et al., 1994; Wikoff et al., 2009; Yano et al., 2015; Kelly et al., 2016; Sampson et al., 2016).

## Microbiota and microbial metabolites affecting the serotonergic system

Food animals, along with humans, have diversity in their intestinal microbiota that is mainly influenced by the surrounding environment and diets and thus share common microbes. These microbes and their hosts have a close relationship surrounding how metabolism occurs for mutualistic or detrimental benefit, depending on the microbial metabolic activity happening in which part of the host gut (Apajalahti, 2005). Different studies have highlighted some influence of gut microbes and their metabolites on the host’s serotonergic system through tryptophan metabolism, serotonin metabolism, and the kynurenine and indole pathway. Among these metabolites, microbial degradation and fermentation product SCFAs are major metabolites produced in the hind gut of avian species (Józefiak et al., 2004). SCFAs have been considered for maintaining gut health of poultry (Liu et al., 2021). The rapid absorption of SCFAs in the hind gut (Ruppin et al., 1980), the association of SCFAs with the BBB (Gerhart et al., 1997; Li et al., 2016), the neuroimmunoendocrine regulatory function of SCFAs (Wikoff et al., 2009; Clarke et al., 2013; Matsumoto et al., 2013) and the neuroprotective effect of SCFAs (Kim et al., 2007) indicate SCFAs to be metabolites important to study for the serotonergic system and overall body.

### Short chain fatty acids

SCFAs, also called volatile fatty acids, provide substantial amounts of energy, commonly fulfilling about 10% of human caloric needs and about 8% of the caloric needs of chicken (Annison et al., 1968). SCFAs in addition modulate the physiology and behavior of animals in various ways. Major SCFAs include acetate (C2), propionate (C3), and butyrate (C4) which are produced in animals through the fermentation of various complex carbohydrates such as dietary fibers, resistant starch, and endogenous substance-like mucins (Annison et al., 1968; Langhout and Schutte, 1996; Józefiak et al., 2004; Sun et al., 2021). The proportion of acetate, propionate, butyrate in the colons of herbivorous animal species ranges from 75:15:10 to 40:40:20 (Bergman, 1990). The cecum is the primary site of microbial fermentation in chickens (Marounek et al., 1999). This is evident by the germ free birds cecum having traces of SCFAs compared to conventional bird however, similar quantities of acetate were found in the peripheral blood of conventional and germ-free birds that demonstrate endogenous source of SCFAs, rather than microbial origin (Annison et al., 1968; Høverstad and Midtvedt, 1986). SCFAs production is advantageous to the host as it is known to improve gut health via maintaining intestinal barrier integrity and immune homeostasis (Furuse et al., 1991; Hu and Guo, 2007; Sunkara et al., 2012; Liu et al., 2021). SCFAs also have been found to inhibit growth of *Salmonella* (Van Immerseel et al., 2003), promote the body weight of broiler chickens (Leeson et al., 2005), and modulate inflammation and oxidative stress (Li et al., 2017). A germ-free mice study highlighted the role of butyrate in improving blood-brain barrier integrity which ensures controlled exchange of biological substances essential for brain activities (Braniste et al., 2014). SCFAs are produced by many bacteria through the glycolytic pathway but there are some varieties, such as *Bifidobacterium* spp., that can produce SCFAs via the pentose phosphate pathway (Macfarlane and Macfarlane, 2003; Cronin et al., 2011). Several bacterial varieties from the *Firmicutes* phylum include butyrate producing *Ruminococcaceae*, *Lachnospiraceae,* and clostridial varieties. *Bacteroides* and *Bifidobacterium* spp. are involved in acetate production. Table 2 shows some of the important studies that have detailed SCFAs with chicken gut bacteria.

**TABLE 2:** Selected studies of SCFA-producing gut microbes in chickens.

Bird sp. and region of isolation	Type of SCFA	Gut microbes identified	References
Broiler chicken cecal-4 weeks old	Butyrate	*Butyricicoccus pullicaecorum* (a *Firmicutes* clostridial cluster IV)	Eeckhaut et al. (2008)
Broiler chicken cecal 6 weeks old	Butyrate	*Faecalibacterium prausnitzii*	Meimandipour et al. (2010)
Broiler chicken cecal 4 weeks old	Butyrate	Isolates of clostridial cluster IV related to *Flavonifractor plautii, Pseudoflavonifractor capillosus, Subdoligranulum variabile, Eubacterium desmolans* and *Butyricicoccus pullicaecorum,* cluster XIVa isolates related to *Anaerostipes caccae, Eubacterium hallii, Clostridium populeti* and *Anaerostipes butyraticus,* cluster XVI related *Eubacterium tortuosum, Eubacterium cylindroides, Streptococcus pleomorphus*	Eeckhaut et al. (2011)
Broiler chickens, ileal mucosa, 3 weeks old	Butyrate propionate	Related to *Enterococcus cecorum* (butyrate) *Butyrivibrio, Coprococcus* (butyrate) *Paludibacter* (propionate)	Shang et al. (2018)
White leghorn chicken caeca	Butyrate	*Megasphaerastantonii* sp. Nov. from genus *Megasphaera*	Maki & Looft (2018)
Cobb 500 broiler chicken, ileal, cecal, 6 weeks	Butyrate	*Ruminococcus, Anaerostipes,* and *Lachnospiraceae*	Jacquier et al. (2019)
Layer chickens, cecal, 8, 20, 50 weeks old	Butyrate Propionate Acetate	Genus *Alistipes* (*Bacteroidetes*) 8 weeks- *Anaerostipes* (butyrate), *Bacteroides thetaiotaomicron* (acetate, propionate) 20 &50 weeks—*Phascolarctobacterium* (propionate) 20 weeks—genus *Bifidobacterium* (acetate)	Sun et al. (2021)

### Short chain fatty acids and the serotonergic system

**FIGURE 1:**
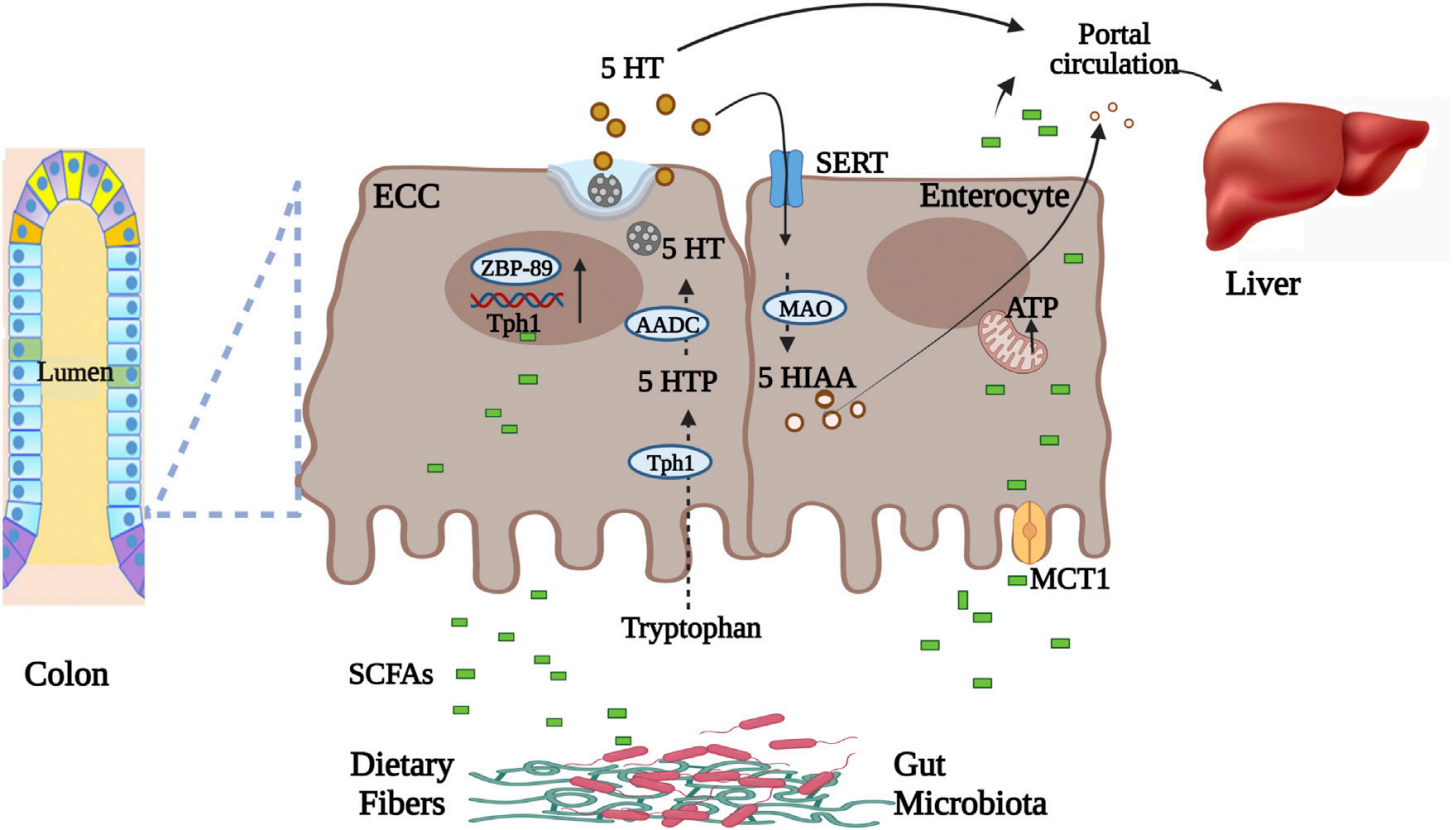
Microbial metabolite SCFAs transportation and role in gut serotonin production. Undissociated form of SCFAs in gut lumen diffuse through enterocytes while dissociated is transported through MCT1 into the circulation. Intestinal enterochromaffin cells synthesize serotonin from tryptophan using Tph1 enzyme. SCFAs in gut lumen stimulate Tph1 expression via zinc finger transcription factor. Secreted serotonin, before entering circulation, is either utilized in the liver or metabolized by enterocytes to 5-HIAA. Part of luminal SCFAs is utilized for energy production by enterocytes. Abbreviations: Enterochromaffin cells (ECC), serotonin (5-HT), zinc finger transcription factor (ZBP-89), tryptophan hydroxylase 1 (Tph1), 5-hydroxytryptophan (5-HTP), amino acid decarboxylase (AADC), monocarboxylated transporter 1 (MCT1), serotonin reuptake transporter (SERT), monoamine oxidase (MAO), hydroxyindoleacetic acid (5-HIAA), short Chain Fatty Acids (SCFAs) (Ritzhaupt et al., 1998; Bloemen et al., 2009; Essien et al., 2013; Reigstad et al., 2015). Figure created with BioRender.com.

SCFAs produced in the gut lumen (undissociated form) diffuse through colonocytes or (dissociated form) transported by monocarboxylated transporters such as monocarboxylated transporter 1 (MCT1, a type of pH-dependent hydrogen-coupled monocarboxylated transporter) and sodium-coupled monocarboxylate transport (SMCT1) (Ritzhaupt et al., 1998) (Figure 1). These SCFAs are metabolized by colonocytes for energy production while unutilized SCFAs undergo hepatic portal circulation (Bloemen et al., 2009). From there, SCFAs are taken up by hepatocytes where they are metabolized for energy or utilized for biosynthesis. Thus, a small portion of SCFAs enters peripheral circulation. In circulation SCFAs interact with different host proteins that include G protein-coupled receptors (GPR41, GPR43, GPR109A) on different tissues (Müller et al., 2019). SCFAs (mainly butyrate) in gut lumen stimulate Tph1 expression in enterochromaffin cells. This then leads to increased production of 5-HT by the enterochromaffin cells (Reigstad et al., 2015). Butyrate elevates Tph1 expression through a butyrate inducible zinc finger transcription factor ZBP-89 (Essien et al., 2013). SCFAs in colonocytes, through varying signaling pathways, influences inflammation by inhibiting NFkB transcription factor), cellular differentiation and proliferation essential for maintaining intestinal homeostasis (Venegas et al., 2019).

**FIGURE 2:**
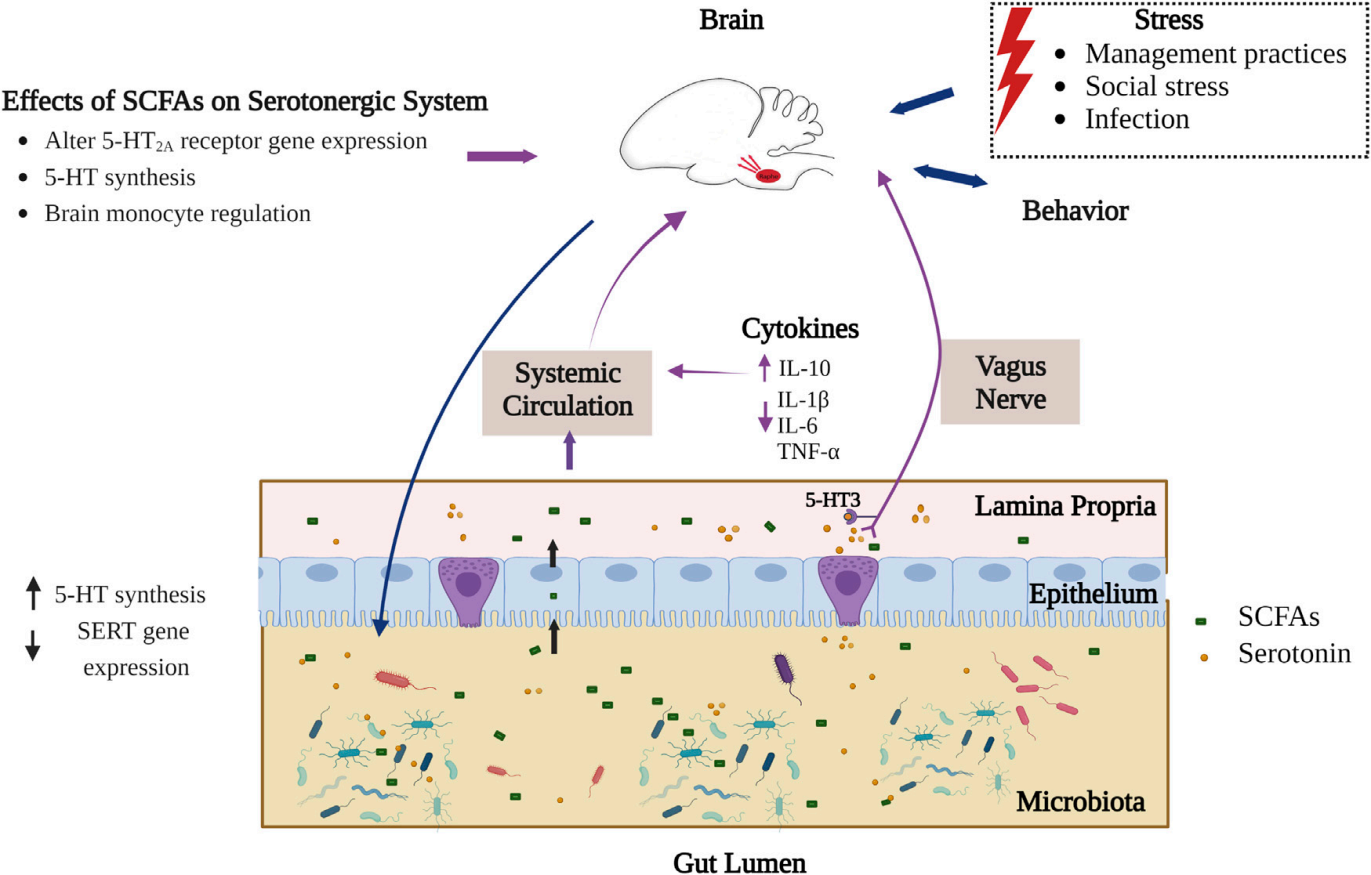
Interaction of SCFAs and serotonergic system in the gut-brain axis. Part of the SCFAs produced in gut lumen interact with the central serotonergic system directly (crossing intestinal and blood-brain barrier) by epigenetic modulation and via activating extrinsic primary afferent vagus nerve (interaction of SCFAs through FFAR3). Serotonin is synthesized by both enterochromaffin cells of the gut epithelium and by gut microbiota. SCFAs also stimulate intestinal serotonin synthesis whereas extracellular serotonin binds to 5-HT3 receptors on afferent vagus nerve and communicates signals to the CNS. On the other hand, different external stressors affect gut microbial composition in birds through the HPA axis and influences production of microbial metabolites like SCFAs. The blue arrows indicate established connection in birds while the violet arrows indicate connections known to occur for some animals but not yet identified in birds (Liu et al., 2012; Sealy and Chalkley, 1978; Yamawaki et al., 2012; Huuskonen et al., 2004; Cook et al., 2021; Gill et al., 2013; Essien et al., 2013; Calefi et al., 2016; Noguera et al., 2018; Gershon and Tack, 2007; Meyer et al., 2012). Figure created with BioRender.com.

The mechanism through which circulatory SCFAs influence the serotonergic system is not fully elucidated and has mainly been investigated with respect to human and mouse models. Considering the very short half-life of SCFAs (such as has been found for butyrate in the bloodstream due to uptake by peripheral tissues), there may be only a minimal concentration of SCFAs that reach the brain when crossing the blood-brain barrier (BBB) (Cummings et al., 1987; Daniel et al., 1989; Mitchell et al., 2011). Within the brain, SCFAs affect brain functioning through direct interactions with G protein-coupled receptors (GPCR) like FFAR2 and FFAR3 (varieties of free fatty acid receptors) (Figure 2). These GPCRs are found in both CNS and peripheral system and are most dense in peripheral organs (Lagerström et al., 2006; Meslin et al., 2015). SCFAs also communicate with the brain via the afferent vagus nerve, leading to the activation of neurons in the CNS area (De Vadder et al., 2014). However, the type of interaction of SCFAs with the vagus nerve, being direct or indirect, is unknown. A study of the vagus nerve FFAR3 knockout mice model showed that SCFAs receptor FFAR3 on the vagus nerve is essential to regulate feeding behavior in animals (Cook et al., 2021). The presence of FFAR3 in the vagus nerve and its influence on feeding behavior may indicate the possibility of SCFA mediated signaling to the central serotonergic system. Additionally, FFAR3 plays an important role in propionate-mediated signals to peripheral and CNS areas for intestinal gluconeogenesis and enhanced noradrenaline secretion by sympathetic neurons respectively (Kimura et al., 2011; De Vadder et al., 2014). Synaptic levels of both neurotransmitters noradrenaline and serotonin are responsible for depressive behavior (Thor et al., 2007). More investigation is overall needed to reveal interactions of SCFAs with the serotonergic system, and the degree to which these interactions may be present and consistent across different varieties of animals, including birds.

Another way by which SCFAs affect the serotonergic system is in their regulation of tryptophan synthesis. As stated earlier, tryptophan is the only precursor for serotonin biosynthesis and its circulating levels depend on dietary intake and gut bacterial tryptophan metabolism (Fernstrom and Wurtman, 1971). Most of the free tryptophan in blood is utilized by the kynurenine (KYN) pathway. Remaining tryptophan has to pass through the BBB for central serotonin synthesis (Peters, 1991). The systemic level of tryptophan is closely linked with inflammation. As proinflammatory cytokines can induce metabolic enzymes like indoleamine 2,3-dioxygenase (IDO) and Tryptophan-2,3-dioxygenase (TDO) involved in KYN synthesis from tryptophan metabolism (Wirleitner et al., 2003; Hestad et al., 2017). Thus, systemic inflammation can limit availability of tryptophan for serotonin synthesis. However, SCFAs in systemic circulation are known to lower the proinflammatory cytokines (TNF-α, IL-1β, IL-6) and elevate anti-inflammatory and regulatory cytokines such as IL-10 which may indirectly increase availability of tryptophan for serotonin synthesis by balancing the cytokines (Liu et al., 2012; Piazzon et al., 2016).

### Short chain fatty acids and histone deacetylase-mediated epigenetic modulation

SCFAs contribute to epigenetic modulation through interaction with histone deacetylases (HDACs) in the brain (Figure 2), however this research has mainly been carried out in mammals. HDACs are crucial in histone deacetylation, which limits the accessibility of genetic material to transcription by compacting chromatin and thus plays an essential role in gene expression (Turner, 2000). HDACs and their regulation are essential for brain development and are studied for neuropsychiatric diseases (Volmar and Wahlestedt, 2015). SCFAs such as butyrate can inhibit HDAC, leading to hyperacetylation resulting in increased accessibility of genes for transcription (Sealy and Chalkley, 1978; Chriett et al., 2019). Monoaminergic neurons, including serotonergic and neuropeptidergic neurons in the brain hypothalamus, express HDACs that deacetylate nuclear as well as cytoplasmic proteins (Takase et al., 2013). Inhibitory effects of butyrate on HDACs have been investigated for serotonin receptor 5-HT2A which are densely present in CNS and high in the cerebral cortex. A gene expression study in sodium butyrate-administered rats has shown downregulation of the 5-HT2A receptor potentially due to inhibitory action of butyrate on HDAC leading to an antidepressant outcome in rats (Yamawaki et al., 2012). Another *in vivo* study on intestinal epithelial cells has further implicated SCFAs with epigenetic change and has shown there to be an inhibitory role of butyrate on HDAC2 that regulates SERT gene expression. Intestinal SERT is essential in maintaining extracellular serotonin levels (Gill et al., 2013). SCFAs have in addition been investigated for brain histone crotonylation as an epigenetic modification that involves transfer of a crotonyl group to lysine residues which influences the gene expression (Tweedie-Cullen et al., 2012), but the functional role of this crotonylation is still unknown (Fellows et al., 2018).

### Short chain fatty acids and neuroinflammation

An understanding of neuroinflammation and the role of short-chain fatty acids in chickens awaits further study. A general understanding would for now involve dynamics as reported for other types of organisms. Butyrate in particular has been found to improve CNS neuroinflammation in mice models induced by lipopolysaccharides (LPS) (Wang et al., 2018; Yamawaki et al., 2018). Neuroinflammation is characterized by activating microglial cells (immune cells of CNS) that follow the elevation of proinflammatory cytokines like IL-6 and TNF-α. At the same time, cytokines and their signaling pathways affect serotonin synthesis and metabolism (Jeon and Kim, 2017). Butyrate can improve circumstances of neuroinflammation through suppression of NF-κB activation and through its aforementioned role in HDAC inhibition, overall controlling the number of microglia cells and astrocytes as has been found in both *in vitro* and *in vivo* models (Huuskonen et al., 2004). These neuroprotective effects of butyrate are observed to enhance memory and restore cognitive functions in mice after systemic or local administration of sodium butyrate (Ferrante et al., 2003; Govindarajan et al., 2011). SCFAs also play a crucial role in immune cell maturation and differentiation. In particular, it has been proposed that SCFAs might regulate brain monocytes such as Ly6Chi, which has been proposed to be essential for hippocampal neurogenesis and memory retention. These monocytes are important for maintaining brain homeostasis (Möhle et al., 2016).

## Discussion

Research on the gut-brain axis has been increasingly extensive in the last decade, stemming from its importance in health and disease, and in maintaining physiological homeostasis. This axis is proving to be particularly important to neurodevelopment and neuropsychiatric disorders. The advancement and availability of sequencing technology has led to a plethora of studies investigating how the gut microbiome plays a major role in the gut-brain axis. The dynamic across this axis regarding the effect gut microbial composition with conditions of the brain has been shown to be influenced by multiple factors, including diet, age, and stress.

Chicken microbiome studies include mostly 16S rRNA gene amplicon sequencing-based studies, but there have been some metagenomics approaches as well (Gilroy et al., 2021). Microbial compositional results of similar chicken breeds have shown variation that can be attributed to experimental protocol or differences between individual chickens (Borda-Molina et al., 2018). Most chicken gut microbiome studies of the gut-brain axis are limited to gut microbial modulations that do not identify underlying mechanisms, such as those possibly involving metabolites. Further research regarding chicken gut microbial metabolites is needed to elevate our knowledge to a level comparable to studies of humans and other common animal models such as mice.

Both for agribusiness and translational objectives, further investigations of the chicken gut-brain-microbiome axis would be well-warranted. Previous studies in chicken have shown bird behavior relating to broad-ranging differences in gut microbiota (Meyer et al., 2013b; Ji et al., 2019). Current findings suggest that some of this dynamic can be circular. Gut microbes potentially influence the serotonergic system and FP behavior in chickens (de Haas and van der Eijk, 2018). Conversely however, feather ingestion also by itself alters gut composition and SCFAs production (Meyer et al., 2012). For how FP continues to pose economic and animal welfare problems, investigating gut microbial metabolites’ effect on the serotonergic system and chicken behavior such as FP and vice versa would be essential for identifying exact mechanisms and associated interventions.

In the case of the translational potential of gut-brain axis research, animal models have helped to reveal the connection between gut microbes and their metabolites with brain neural processes and functioning. Microbiome, behavioral, serotonin and other physiologic indicators implicate similar dynamics across these two different organisms. Compared to chickens, while some other animal models have helped illuminate methodologies and general findings of gut-brain axis dynamics, their translational value can be limited. The germ-free mouse model has enriched gut-brain axis research, showing for instance that cognitive deficits that can be restored on microbiota transplantation (Luczynski et al., 2016). Current clinical beneficial effects of microbiota transplantation have been limited to treating irritable bowel syndrome (IBS). The possible reasoning behind this limited translational impact thus far may relate to the constrained range of animal models that have been utilized. The detachment of laboratory mice from the natural environment means that these models lack the environmental exposure similar to humans and thus lack gut microbial diversity (Masopust et al., 2017). By comparison, chickens can be readily studied in outdoor and indoor environments through commonly available agricultural enclosures. Past research on avian cognitive neuroscience has furthermore found that the avian brain can be used to understand human cognition despite significant physiological and genetic differences (Rose, 2000; Clayton and Emery, 2015). Domestication of chicken by humans and similarity in the microbial community at higher taxonomic levels supports logistics and relevance for how chickens as a model animal can be used to investigate the gut-brain axis with the hope of high translational efficiency (Kohl, 2012). The similarity in microbial community and complexity facilitates further development and calibration of underlying biotechnological and analytical methodologies needed for robust examinations of microbiomes. Finally, as is the case with other vertebrates, the chicken GI tract may be considered to enclose diverse microbiota and their metabolites, with some of these metabolites being modulators of birds’ behavior. Gut microbial metabolites SCFAs stimulate enteric serotonin synthesis and are responsible for maintaining gut health. SCFAs affect brain functioning through direct interaction via HDAC-mediated epigenetic modulation and immune signaling. A challenge remains however with most of these studies being from mice models. Further mechanistic and longitudinal studies in chickens would help validate the likely consistency by which these mechanisms dynamics could be considered across animals in general, including humans. There is overall joint benefit for how further research into SCFAs within chickens helps to advance chickens as a model animal to be considered further for translational and applied gut-brain axis studies, as would both help tackle complex, multifaceted neuropsychiatric disorders in humans and investigate conditions of health and behavior of chickens in agricultural contexts.

## Conclusion

Previous research studies in avian species have shown that experimental manipulation of gut microbiota has an impact on bird behavior. There is a wide range of behaviors that are influenced in birds that includes FP which is considered important for poultry welfare. However, there are fewer studies in birds investigating exact mechanisms that drive the gut-microbiome-brain axis. Chicken gut microbiota have a high abundance of *Bacteroidetes* and *Firmicutes* phyla which includes most of those bacterial genera that produce SCFAs. SCFAs and serotonin are important mediators of the gut-microbiome-brain axis with, for an instance, the influence of SCFAs on peripheral as well as central serotonergic systems and the potential association of serotonin with FP behavior in birds. Chicken gut microbial metabolites like SCFAs and their effects on the serotonergic system remain an essential area for further inquiry needed to understand behavioral outcomes in birds. Considering the nature of SCFAs interactions and the conserved molecular and behavioral attributes of the serotonergic system, poultry chicken may be an emergent translational model for identifying underlying mechanisms of change within the gut-microbiome-brain axis.
